# Spontaneous Rupture of an Adrenal Artery in Pregnancy: A Case Report

**DOI:** 10.1155/2012/859068

**Published:** 2012-12-30

**Authors:** D. Bolla, V. Schyrba, G. Drack, S. Dietler, R. Hornung

**Affiliations:** Department of Obstetrics and Gynecology, Cantonal Hospital of St. Gallen, Rorschacher Straß 95, 9007 St. Gallen, Switzerland

## Abstract

A spontaneous rupture of an adrenal artery is a rare cause of abdominal pain in pregnancy. We present a case of a pregnant woman who needed to be operated on because of a rupture of the right adrenal artery associated with a fetal bradycardia. An immediate caesarean section was performed. The intra-abdominal palpation identified an extensive retroperitoneal mass near the right kidney and a postoperative computer tomography confirmed an active bleeding near the kidney. For this reason our interventional radiology team, using a right femoral artery approach, performed a flush aortogram and identified the source of bleeding in the right adrenal artery. After two attempts, a coiling of the artery stopped the haemorrhage. The pathogenesis of arterial haemorrhage is still poorly understood although a possible cause could be the excess of hormones during pregnancy, which can lead to a significant arterial wall degeneration. In case of a retroperitoneal bleeding and if the patient is still haemodynamically stable, a transcatheter embolization using microcoils must be considered. This technique is nowadays safe and effective and can be performed within a short time with a lower risk of complications.

## 1. Introduction

A spontaneous rupture of an adrenal artery is extremely rare in pregnancy. In the literature, only a few cases of rupture of an adrenal artery have been reported [1–3]. The frequency, in nonpregnant women, varies from 0.03% to 1.8% of all visceral aneurysms [2]. The most common localization of visceral aneurysms is the celiac followed by mesenteric, hepatic, and splenic arteries [4].

We present a further case of a spontaneous rupture of the right adrenal artery, which was detected after a caesarean delivery performed because of a fetal bradycardia. 

## 2. Case

A 28-year-old gravida 1 with an uneventful course of pregnancy and no history of a trauma was referred to our hospital because of an unclear retroperitoneal mass. This was diagnosed at a nearby hospital where the patient had presented with an acute right-sided abdominal pain at 32 1/7 weeks of gestation. By admission in the referral hospital, she had an unremarkable medical history without evidence of trauma. There was no vaginal bleeding or uterine activity, but fetal bradycardia was detected. An immediate caesarean section was performed under general anaesthesia. A female newborn, weighing 1970 g, was delivered with an Apgar score of 0 at 1 minute, 1 at 5 minutes, and 5 at 10 minutes, respectivly. The pH from the umbilical artery was 6.88. The placenta showed no signs of haemorrhage. The intra-abdominal palpation identified an extensive retroperitoneal mass near the right kidney. With a suspicion of a retroperitoneal haemorrhage, the operation was interrupted and the patient was transferred intubated and after transfusion of 3 units of red cells to our hospital. At admission, an abdominal ultrasound and a subsequent computer tomography confirmed an active bleeding near the kidney. For this reason our interventional radiology team, using a right femoral artery approach, performed a flush aortogram and identified the source of bleeding in the right adrenal artery. After two attempts, a coiling of the artery stopped the haemorrhage. Following this, the patient stabilized haemodynamically and two days later, the clothed blood mass was removed laparoscopically. The postoperative course was uncomplicated and the patient was discharged 10 days after the first intervention.

## 3. Discussion

Pregnancy is considered an important risk factor for aneurysm. Fifty percent of arterial ruptures occur in young multiparous pregnant women preferentially in the third trimester or in the postpartum period. The reason is a significant arterial wall degeneration due to the influence of the pregnancy related hormones on the smooth muscle cells of the arterial wall [1].

This arterial wall deterioration associated with the haemodynamic changes during pregnancy can produce an aggravation or formation of aneurysms increasing the risk of a spontaneous arterial dissection [5]. Other risk factors not pregnancy related are, for example, trauma, sepsis, haematologic disorders, underlying adrenal tumours, stress, arteriosclerosis, and congenital malformations. 

Visceral aneurysms are generally asymptomatic, but if they rupture the patients typically present with acute abdominal, flank or back pain, nausea, and vomiting associated with haemodynamic instability [4]. In late pregnancy, haemorrhagic shock with no specific pain is often confused with other diseases like abruption of the placenta or ruptured uterus. This occurred also in our case where the abdominal pain associated with fetal bradycardia was mistaken for a placental abruption. Depending on the vessel involved, the bleeding can be transiently contained into the abdominal cavity. The appropriate treatment depends on the degree of emergency. During the caesarean section, the retroperitoneal mass caused by the retroperitoneal location haemorrhage could be identified. Thanks to the contained bleeding, the patient could be transferred to our hospital and treated by interventional radiography. With the advantage of more and more sophisticated technologies such as the diagnostic ultrasound and the computed tomography, an initial diagnostic suspicion associated with the degree of urgency can be verified in a few minutes. This is very important in choosing the type of treatment. 

If the bleeding is free into the abdomen and the patient is instable, an emergency surgery is indicated. In these cases, the risk of not to identify intraoperatively the point of bleeding at the time of initial laparotomy is high and the maternal and fetal mortality increases significantly (30%) [6]. The operation consists in stopping the bleeding by ligature of the artery proximally to the aneurysm and subsequently the removal of the haematoma. This procedure is not simple especially if the patient is instable with signs of shock. 

The conservative approach, on the contrary, is indicated for those where bleeding is contained and the patient is haemodynamically stable. In our case the interventional radiologist was called and an arteriography was immediately performed. The ruptured adrenal artery was identified within a relatively short space of time and it was then possible to coil the artery and stop the bleeding directly, without compromising other larger vessels and allowing at the same time the preservation of functional adrenal parenchyma.

## 4. Conclusion

Rupture of an adrenal artery aneurysm is a rare but serious clinical entity that requires in most of the cases an emergency surgery. In selected cases, where the patient is still haemodynamically stable, a transcatheter embolization using microcoils must be considered. This technique is nowadays safe and effective and can be performed within a short time with a lower risk of complications.
